# EGF-mediated EGFR/ERK signaling pathway promotes germinative cell proliferation in *Echinococcus multilocularis* that contributes to larval growth and development

**DOI:** 10.1371/journal.pntd.0005418

**Published:** 2017-02-27

**Authors:** Zhe Cheng, Fan Liu, Xiu Li, Mengya Dai, Jianjian Wu, Xinrui Guo, Huimin Tian, Zhijie Heng, Ying Lu, Xiaoli Chai, Yanhai Wang

**Affiliations:** 1 State Key Laboratory of Cellular Stress Biology, School of Life Sciences, Xiamen University, Xiamen, Fujian, China; 2 Parasitology Research Laboratory, School of Life Sciences, Xiamen University, Xiamen, Fujian, China; 3 Medical College, Xiamen University, Xiamen, Fujian, China; University of Würzburg, GERMANY

## Abstract

**Background:**

Larvae of the tapeworm *E*. *multilocularis* cause alveolar echinococcosis (AE), one of the most lethal helminthic infections in humans. A population of stem cell-like cells, the germinative cells, is considered to drive the larval growth and development within the host. The molecular mechanisms controlling the behavior of germinative cells are largely unknown.

**Methodology/Principal findings:**

Using *in vitro* cultivation systems we show here that the EGFR/ERK signaling in the parasite can promote germinative cell proliferation in response to addition of human EGF, resulting in stimulated growth and development of the metacestode larvae. Inhibition of the signaling by either the EGFR inhibitors CI-1033 and BIBW2992 or the MEK/ERK inhibitor U0126 impairs germinative cell proliferation and larval growth.

**Conclusions/Significance:**

These data demonstrate the contribution of EGF-mediated EGFR/ERK signaling to the regulation of germinative cells in *E*. *multilocularis*, and suggest the EGFR/ERK signaling as a potential therapeutic target for AE and perhaps other human cestodiasis.

## Author summary

*E*. *multilocularis* is considered as one of the most lethal parasitic helminth in humans. It grows like tumors mainly in human liver and infiltrates other tissues, and even metastasizes. It is believed that the parasite possesses a population of stem cell-like cells, the germinative cells. These cells are totipotent, have the ability for extensive self-renewal, and drive the parasite’s development and growth in the host. However, mechanisms controlling the behavior of germinative cells are poorly understood. Here, we show that the highly conserved EGFR/ERK signaling pathway in the parasite promoted germinative cell proliferation upon addition of human EGF (epidermal growth factor) *in vitro*, resulting in stimulated growth and development of the parasite. Our study provides information important for understanding this mechanism regulating germinative cells and the complex host-parasite interaction, and we hope it will help in developing new therapeutic strategies for the treatment of human helminthic infections.

## Introduction

A population of pluripotent adult somatic stem cells, well known as “neoblasts”, has been extensively characterized and documented for free-living platyhelminthes (flatworms) [1–5]. Neoblasts represent the only proliferative cell population, responsible for cell renewal during homeostasis, development and regeneration [6–11]. Like in its free-living relatives, neoblast-like stem cells and similar cell renewal mechanisms also exist in the other two main groups of flatworms, trematodes (flukes) and cestodes (tapeworms), both living a parasitic life [3, 12–15]. In cestodes, a population of undifferentiated cells, called “germinative cells”, is the only source for cell proliferation. These germinative cells are totipotent and are thought to drive growth and development throughout the life cycle of cestodes [12, 15–16].

The larval stage of the cestode *Echinococcus multilocularis* is the causative agent of alveolar echinococcosis (AE), one of the most lethal human helminthiasis [17]. An infection is initiated when the intermediate host (rodents, humans) ingests infective eggs produced by adult tapeworms. The oncosphere hatches from the egg and then develops in the liver into cyst-like metacestode vesicles, which grow like tumors and infiltrate host tissue, forming new vesicles and even metastasizes. The metacestode vesicles bud giving rise to brood capsules, which in turn generate protoscoleces by asexual multiplication. Protoscoleces can either mature into adult tapeworms if ingested by the definitive host (canids) or develop into metacestode vesicles when distributed in the intermediate host. This unique proliferation potential of *E*. *multilocularis* metacestode larvae is believed to be based upon the germinative cells, which are totipotent and have the ability for extensive self-renewal [15].

It has been well documented that the maintenance of pluripotency and self-renewal capacity of stem cells requires a continuous input from cell-extrinsic signals [18–19]. The extrinsic factors initiate various intrinsic signaling cascades which in turn maintain stem cells and regulate their functions. Signaling axes including LIF/gp130/STAT3, BMPs/BMPRs/Smads, Wnt/Frizzled/β-catenin, PI3K/AKT, and FGF/FGFR have been extensively evidenced to participate in controlling the survival, self-renewal, and differentiation of stem cells [19–20]. Increasing evidence has shown that the epidermal growth factor receptor (EGFR)-dependent signaling pathways also play important roles in the maintenance and regulation of stem cells [21–25].

The metacestode larvae of *E*. *multilocularis* grow and proliferate in close contact with the intermediate host’s tissues, mainly within the liver. The microenvironment for metacestode development involves a number of host-derived hormones and cytokines, such as insulin, bone morphogenetic protein (BMP), fibroblast growth factor (FGF) and epidermal growth factor (EGF) [26]. These host-derived factors are thought to bind to parasite receptors and in turn influence the parasite’s growth and development through exhibiting their impacts on the relevant evolutionarily conserved signaling systems within the parasite [26–27]. A recent study has evidenced that host insulin activates *E*. *multilocularis* PI3K/AKT signaling pathways and stimulates germinative cell proliferation and larval development [28]. In addition, *in vitro* cultivation of *E*. *multilocularis* larvae and primary cells requires continuous presence of host cells as the feeder cells (like stem cell cultivation) or host cell-conditioned medium which contains host-derived growth factors [29]. Together, lines of evidence offer compelling clues that the conserved signaling pathways in *E*. *multilocularis* could respond to host factors and may regulate germinative cells which are fundamental for the larval growth and development of the parasite [30].

It has been shown that the Ras/Raf/MEK/ERK signaling in *E*. *multilocularis* is activated in response to a host-derived EGF signal, which is most probably mediated by the parasite’s EGFR-like kinase [31–33]. Using *in vitro* cultivation systems we show here that *E*. *multilocularis* EGFR/ERK signaling pathway is activated upon addition of human EGF and promotes germinative cell proliferation during the parasite’s larval growth and development.

## Methods

### Ethics statement

All animal experiments were conducted in strict accordance with China regulations on the protection of experimental animals (Regulations for the Administration of Affairs Concerning Experimental Animals, version from July-18-2013) and specifically approved by the Institutional Animal Care and Use Committee of Xiamen University (Permit Number: 2013–0053).

### Parasite in vitro culture, growth and development assay, and treatment

The parasite isolate used in this study was obtained from Hulunbeier Pasture of Inner Mongolia of China [34] and maintained by *in vivo* propagation of the parasite material in mice (supplied by Xiamen University Laboratory Animals Center, XMULAC). *In vitro* cultivation of metacestode vesicles was performed using host cell conditioned medium according to a previously established protocol [35] unless otherwise indicated. For the growth assay, vesicles (diameter < 1mm) were manually picked up and cultured in 24-well cell culture plates supplemented with different media as indicated in the text. 100 ng/mL recombinant human EGF (PeproTech, Rocky Hill, NJ) was used for all experiments unless otherwise indicated. Parasite growth was determined by the measurement of vesicle’s diameter under inverted microscope weekly. Each group contains at least 3 replicates and more than 150 vesicles in total for each group were analyzed. Two-three independent experiments were performed. For the treatment of inhibitors, the EGFR inhibitors CI-1033 and BIBW2992 or the MEK inhibitor U0126 (Selleck Chemicals, Houston, TX) was added into the culture medium at a final concentration as indicated. All experiments were performed with exchange of the medium containing the same ingredients every three days. Protoscoleces were collected from parasite material and *in vitro* cultured in conditioned medium. The vesicle formation process, in which protoscoleces dilate and vacuolate, were examined after 18 days of culture. 40 mM of hydroxyurea was used to treat metacestode vesicles as described before [15].

### BrdU incorporation assay, EdU labeling and EdU-BrdU dual labeling

Vesicles were incubated with BrdU for two days and chromosomal DNA was isolated for BrdU incorporation assay with the colorimetric BrdU ELISA kit (Roche, Mannheim, Germany) as described before [28].

Vesicles were incubated with 50 μM of EdU for 4 hours and whole-mount prepared according to Cheng et al. [36]. Click-iT-EdU Alexa Fluor 555 Imaging Kit (Life Technologies, Shanghai, China) was used for detection of EdU.

For the EdU-BrdU dual labeling, vesicles were incubated with 10 μM EdU for 4 hours, washed and then cultured with no labeling for 44 hours. 10 μM BrdU was next used for continuous labeling for another 24 hours. Vesicles were fixed at the end of the labeling period and whole-mount prepared (see also S2C Fig). After a 45-minute 2N HCl treatment, immunofluorescence was performed for BrdU detection using the anti-BrdU antibody (clone MoBU, Life Technologies) followed by EdU detection. DNA was counterstained with 4’, 6-diamidino-2-phenylindole (DAPI) (Sigma, St. Louis, MO) for all labeling experiments.

For the inhibitor experiments during the recovery from hydroxyurea treatment, vesicles were allowed to recover in the conditioned medium supplemented with EGF. 10 μM CI-1033 or 20 μM U0126 was then added into the medium immediately after the initial EdU pulse. Germinative cell proliferation was analyzed by EdU-BrdU dual labeling after 4 days of recovery.

For the quantification of EdU^+^ or BrdU^+^ cells, at least 12 random microscopic fields from 4–6 vesicles were captured and the positive cells were manually counted. 3–5 labeling experiments were performed and analyzed for each control and treatment group.

### mRNA expression analysis of *E. multilocularis* EGFR members

Total RNA was extracted from *in vitro*-cultivated protoscoleces or metacestode vesicles, treated with RNase-free DNase and reverse transcribed into cDNA. cDNAs were processed for RT-PCR analysis using the primers: EmER-qF2 (5’-GCG AAT GTA AGC ATT TCA AGT CA-3’) and EmER-qR2 (5’-TTC ACA AAG TAG CAG AAA GCA CAT-3’) for *Emer*; 617300-qF (5’-GCC GCA TCT ATG GAC ACGC-3’) and 617300-qR (5’-AGT CAT CTT GTG GGA GGA ATCG-3’) for Emuj_000617300; 969600-qF (5’-CTC TGG GGT GTC TGC TGT CC-3’) and 969600-qR (5’-TCC CAC AGA GTC ACA CCG TAGG-3’) for Emuj_000969600.

### Expression of EmER in *Xenopus* oocytes

Expression of the parasite EGF receptor EmER in *Xenopus* oocytes was performed according to [37]. Briefly, the full-length coding sequence of EmER was cloned into the *Xenopus* oocyte expression vector pXT7-flag (a gift from Dr. Li Guang in Xiamen University). Linearized plasmids were then used as the templates for capped mRNA (cRNA) synthesis using the T7 mMessage mMachine Kit (Ambion). Oocytes were obtained from *Xenopus laevis* (supported by Stem Cell Bank, Chinese Academy of Sciences) and then microinjected with EmER cRNA or water (noninjected control). Membrane proteins were extracted from 30 oocytes after 48 h of injection and immunoprecipitated by the anti-flag monoclonal antibody (Sigma) and analyzed by western blot. For EGF and CI-1033 treatment, oocytes that had been expressing EmER for 48 h were incubated with 10 μM of CI-1033 or DMSO for 4 h followed by 20 minutes of EGF stimulation. Membrane extracts were immunoprecipitated and analyzed by western blot using the anti-flag or anti-phospho-tyrosine (CST, Beverly, MA) monoclonal antibodies.

Induction of GVBD (germinal vesicle breakdown) in EmER-expressing *Xenopus* oocytes was performed according to [37]. Oocytes that had been expressing EmER for 48 h were pretreated with 10 μM of CI-1033 or DMSO for 4 h and then incubated with EGF. GVBD was monitored after 16 h of EGF incubation. As a positive control for GVBD, oocytes were stimulated with progesterone (PG), the natural inducer. 20–30 oocytes were used for each group and three independent experiments were performed.

### Western blot and immunofluorescence

Lysates of *in vitro*-cultivated protoscoleces or metacestode vesicles were produced, separated on 12% acrylamide gels and transferred to PVDF membranes. Detection of *E*. *multilocularis* ERK and phosphorylated ERK was performed according to Spiliotis et al. [32] using the polyclonal rabbit anti-ERK1/2 (Stressgen, Victoria, Canada) and anti-ERK1/2 [pTpY^185/187^] (Life Technologies) antibodies, respectively. The anti-rabbit IgG antibody conjugated with horseradish peroxidase (Theromo Scientific, Shanghai, China) was used as a secondary antibody. For all western blot experiments, detection of *E*. *multilocularis* actin was performed using the polyclonal anti-β-actin antibody (CST) as loading controls.

Immunofluorescence was performed using the whole-mount prepared metacestode vesicles as described before [36]. For Histone H3 detection, the anti-phospho-Histone H3 antibody (Ser10, 1:200) (CST) was used. For all immunofluorescence experiments, an Alexa 488-conjugated second antibody (Life Technologies) was used and DNA was counterstained with DAPI.

### Data analysis and statistics

Data of three or more experimental repeats are shown as mean ± SD as indicated in the respective figure legend unless otherwise indicated. The mean values of the data from the experimental groups were compared by performing two-tailed Student’s t-test and the *P* values were indicated as those: *P < 0.05, **P < 0.01, and ***P < 0.001.

## Results

### EGF stimulates *E. multilocularis* larval growth and development

**Fig 1:**
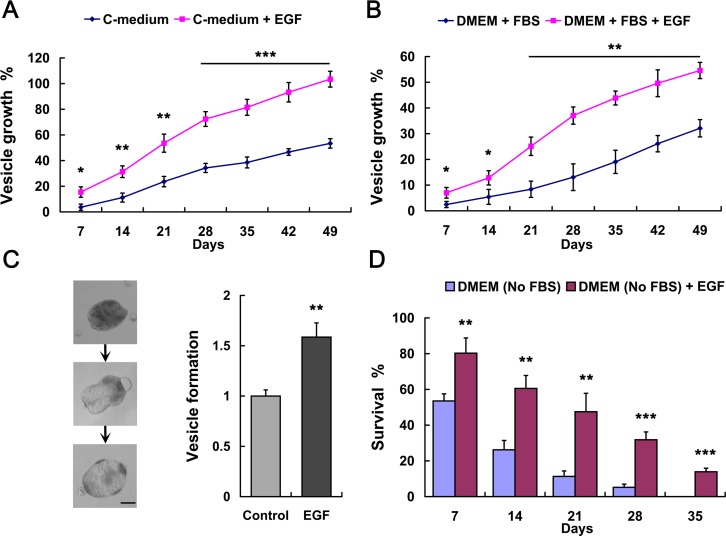
EGF stimulates *E. multilocularis* larval growth and development. (A-B) Metacestode vesicles were cultivated in conditioned medium (C-medium) (A) or serum-containing DMEM (B) supplemented with 100 ng/mL recombinant human EGF or not. Vesicle growth is shown as the increase of vesicle diameter as compared to day 0. Comparison between the EGF group and the control group at the same timepoint was performed using two-tailed Student’s t-test. (C) Vesicle formation from protoscolex in conditioned medium (control) with addition of EGF. Control was set to 1 and results were normalized against the control (right). Representative images of the formation process are shown (left). Bar = 50 μm. (D) Survival of the vesicles in serum-free DMEM supplemented with EGF. Data in (A-D) are shown as mean ± SD of at least three replicates, representative of 2–3 independent experiments.*P < 0.05, **P < 0.01, and ***P < 0.001.

To examine the impacts of EGF on the larval growth of *E*. *multilocularis*, metacestode vesicles were incubated in host cell-conditioned medium supplemented with recombinant human EGF. We found that 10 ng/mL or higher concentrations of EGF can greatly promote vesicle’s growth (S1 Fig). We then used 100 ng/mL of EGF which showed the most significant effect on parasite’s growth *in vitro* for further studies. The results show that addition of EGF can stimulate the growth of metacestode vesicles (Fig 1A). Similar results were observed after EGF was added into the Dulbecco’s modified eagle medium (DMEM) containing 10% serum (Fig 1B). We also found that EGF greatly promoted the vesicle formation process, in which the protoscoleces dilated and vacuolated (Fig 1C). These results illustrate that the larval growth and development of *in vitro*-cultivated *E*. *multilocularis* larvae could be stimulated by exogenously added EGF, which is probably mediated by an EGFR-dependent signaling in the parasite.

Considering that either serum or conditioned medium contains complex ingredients, we then incubated vesicles in the serum-free DMEM only supplemented with EGF. Addition of EGF could not obviously stimulate the growth of vesicles, however, it remarkably promoted their survival (Fig 1D). Although metacestode vesicles could not survive for long in this situation, the method excludes the influence of other host factors in serum and suggests that the parasite is responsive to host EGF stimulation. Further experiments in this study were all performed using host cell-conditioned medium unless otherwise indicated.

### EGF promotes the proliferation of *E. multilocularis* germinative cells

**Fig 2:**
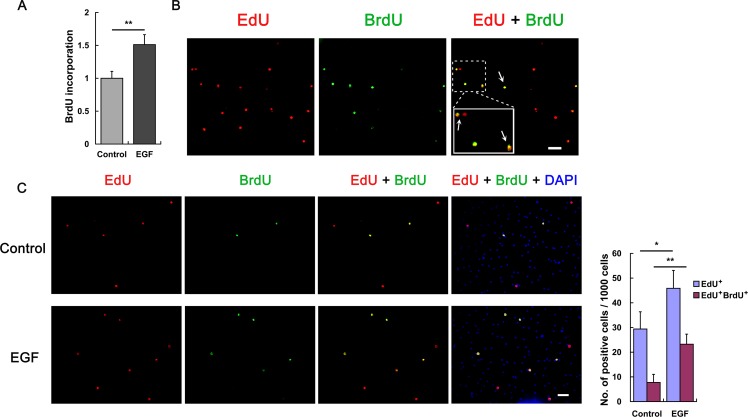
EGF promotes *E. multilocularis* germinative cell proliferation. (A) Vesicles were incubated with BrdU for two days and chromosomal DNA was isolated for BrdU incorporation assay. Control was set to 1 and results were normalized against the control. Data are shown as mean ± SD of triplicates. ** *P* < 0.01. (B) Vesicles were *in vitro* cultivated under normal conditions and germinative cell proliferation was visualized by EdU-BrdU dual labeling. Insert shows the magnified view. Arrows indicate EdU^+^BrdU^+^ cells. Bar = 20 μm. (C) Vesicles were pretreated with 40 mM of hydroxyurea for three days and allowed for recovery in conditioned medium (control) supplemented with EGF. Germinative cell proliferation was analyzed after 4 days of removal of hydroxyurea and representative images are shown. Quantification of EdU^+^ and EdU^+^BrdU^+^ cells is shown in the right panel. Data are shown as mean ± SD of 3 separate labeling experiments. * *P* < 0.05; ** *P* < 0.01. Bar = 20 μm.

Given that the germinative cells, a population of stem cell-like cells, drive larval growth and development of *E*. *multilocularis*, we then investigated the impacts of EGF on the germinative cells. Vesicles were incubated with 5-bromo-2’-deoxyuridine (BrdU), an analogue of thymidine used for studying cell proliferation by detecting its incorporation into the newly synthesized DNA of replicating cells. The result shows that addition of EGF greatly stimulated the BrdU incorporation in the vesicles (Fig 2A). Since germinative cells are the only cells capable of proliferation in metacestode vesicles, this result suggests that EGF promotes germinative cell proliferation.

A dual labeling method through sequential pulses of 5-ethylnyl-2’-deoxyuridine (EdU) and BrdU, which has been utilized to verify the self-renewal capacity of adult somatic stem cells in the human blood fluke *Schistosoma mansoni* [14], was further applied to determine the effects of EGF on germinative cells. EdU is a newly found analogue of thymidine [38], which has been shown to be incorporated by proliferating cells of *E*. *multilocularis* [15].

Under normal *in vitro* cultivation conditions, we found that most of the dividing germinative cells in the vesicles could incorporate EdU before a chase period of 44 h, which was further used for EdU-BrdU dual labeling experiments (S2A Fig). The results show that about 48% of cells that initially incorporate EdU are BrdU^+^ 3 days after an initial EdU pulse (749 EdU^+^BrdU^+^ / 1554 EdU^+^ nuclei, 4 independent labeling experiments) (Fig 2B), indicating that these germinative cells divide and produce proliferation-competent daughter cells that initially incorporate EdU can incorporate BrdU during the second replication.

It has been shown that depletion of the germinative cells in *E*. *multilocularis* vesicles could be achieved through hydroxyurea treatment for longer periods of time (*e*.*g*. seven days) and that the germinative cells undergo clonal expansion like stem cells after removal of hydroxyurea [15]. We also performed similar experiments and found that the EdU^+^BrdU^+^ cells are highly presented in the clonally growing germinative cells (S3 Fig), suggesting that the EdU^+^BrdU^+^ cells are extensively proliferating germinative cells and a part of them might be undergoing self-renewing divisions.

To further investigate the effect of EGF on germinative cells, we treated vesicles with hydroxyurea to eliminate most germinative cells [15]. After removal of hydroxyurea, germinative cell proliferation/self-renewal was allowed for recovery in the medium with addition of EGF or not for 4 days, and the EdU-BrdU sequential pulses began on the second day of the recovery (see also S2B and S2C Fig). At the end of the dual labeling period, the results show that addition of EGF induced significantly more numbers of both EdU^+^ and EdU^+^BrdU^+^ cells in the vesicles compared to the controls (Fig 2C). We found that the proportion of EdU^+^BrdU^+^ cells with respect to the number of EdU^+^ cells was also greatly increased (25.6% and 50.5% for the control and EGF-treated groups, respectively, statistical significance *P* = 0.00101), suggesting increased continuous proliferation, and possibly promoted self-renewal of the germinative cells upon EGF stimulation. Together, these results support the findings that addition of EGF can promote the proliferation of germinative cells (Fig 2A), which subsequently drives the larval growth and development of *E*. *multilocularis*. These results also suggest that an EGFR-dependent signaling in the parasite may be involved in regulating germinative cell proliferation upon EGF stimulation.

### EGFR/ERK signaling contributes to germinative cell proliferation

*E*. *multilocularis* possesses an EGFR-like kinase (EmER) which is suggested to interact with host EGF [31, 33]. Besides EmER, we also found two additional *E*. *multilocularis* EGF receptor members (Emuj_000617300 and Emuj_000969600) by analyzing *E*. *multilocularis* genome sequence (http://www.genedb.org/Homepage/Emultilocularis) and fully cloned and sequenced the respective genes. Comparisons of the putative protein sequences reveal that these EGF receptor members exhibit significant homologies to human EGFR and the EGFR homologue of the closely related schistosome *S*. *mansoni*, especially in the tyrosine kinase domains (S4A–S4D Fig). The results of mRNA expression analysis show that these EGF receptor homologues are constitutively present in *E*. *multilocularis* metacestode vesicles and protoscoleces (S4E Fig).

**Fig 3:**
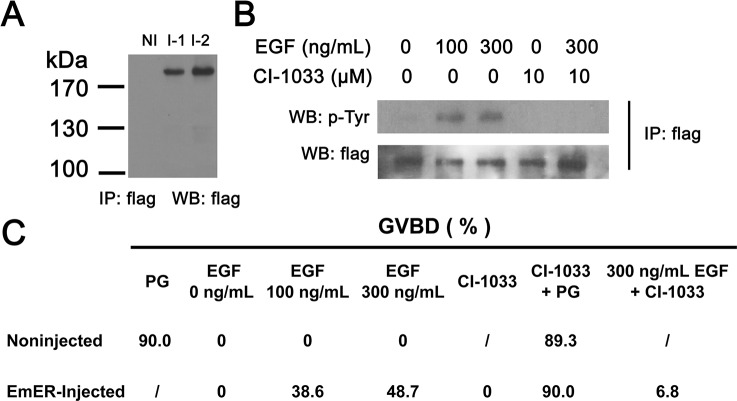
EGF activates *E. multilocularis* EGF receptor EmER in *Xenopus* oocyte expression system. (A) Membrane extracts were prepared from *Xenopus* oocytes expressing EmER or not (NI, noninjected) and immunoprecipitated and analyzed by western blot using the anti-flag antibody. The results of two independent injection experiments are presented (I-1 and I-2). The bands exhibited a molecular mass larger than expected (178 kDa), which could be attributable to glycosylation. (B) Oocytes expressing EmER were incubated with CI-1033 or DMSO for 4 h followed by 20 minutes of EGF stimulation. Membrane extracts were immunoprecipitated by the anti-flag antibody and analyzed by western blot using the anti-flag or anti-phospho-tyrosine antibodies. (C) Induction of GVBD (germinal vesicle breakdown) in EmER-expressing *Xenopus* oocytes. Oocytes that had been expressing EmER for 48 h were pretreated with CI-1033 or DMSO for 4 h and then incubated with EGF. GVBD was monitored after 16 h of EGF incubation and the mean percentages of oocytes exhibiting GVBD for three separate experiments are shown. Noninjected oocytes were used as controls. DMSO (final concentration 0.25%) was found no effects on GVBD. PG: progesterone; “/”: not tested.

To investigate whether the *E*. *multilocularis* EGF receptor(s) respond to EGF stimulation or not, we utilized the *Xenopus* oocyte expression system, which is a powerful tool for receptor tyrosine kinase research and has been successfully used for studying the EGF receptor (SER) in *S*. *mansoni* [37]. The results show that the parasite EGFR EmER could be efficiently expressed in *Xenopus* oocytes with a molecular weight approximately 200 kDa (Fig 3A), and that addition of EGF resulted in the activation of EmER by detection of phosphorylated tyrosine (Fig 3B). It has previously been shown that host EGF could induce germinal-vesicle breakdown (GVBD) in *S*. *mansoni* SER-expressing oocytes [37]. Similar results were also observed in the EmER-expressing oocytes (Fig 3C), suggesting that addition of exogenous EGF could activate the parasite EGFR in the oocytes and induce GVBD. Using the oocyte system, we also found that CI-1033 (canertinib), an irreversible inhibitor for human EGF receptors [39], could effectively inhibit the EGF-induced activation of EmER and GVBD (Fig 3B and 3C).

**Fig 4:**
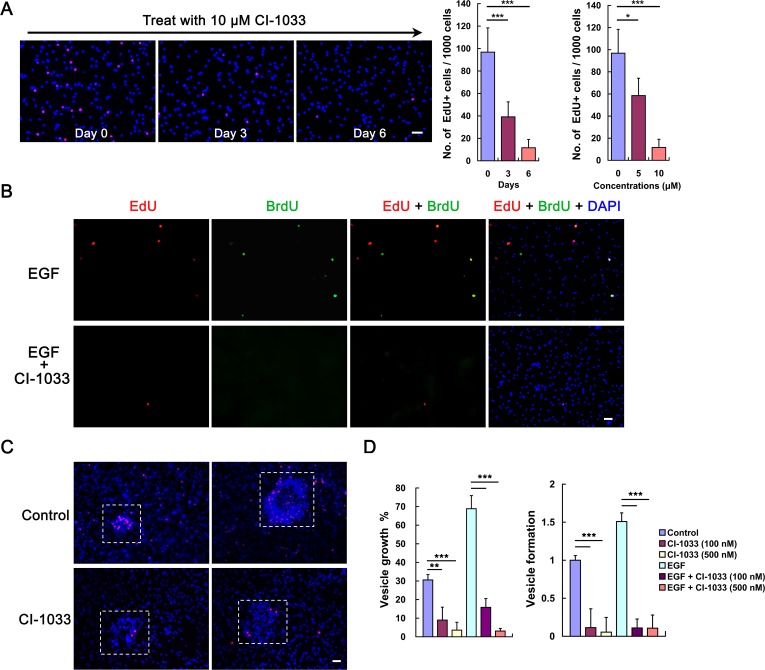
Inhibition of *E. multilocularis* EGFR impairs germinative cell proliferation. (A) Metacestode vesicles were treated with 10 μM CI-1033 and the representative images for day 0, 3 and 6 are shown on the left (red: EdU; blue: DAPI). Quantifications of the EdU^+^ germinative cells in the vesicles treated with 10 μM CI-1033 for indicated time (middle) and 5–10 μM CI-1033 or DMSO control (0) for 6 days (right) are shown. Values represent the mean ± SD of 5 separate labeling experiments. * *P* < 0.05; *** *P* < 0.001. (B) Effects of CI-1033 on germinative cell proliferation during the recovery from hydroxyurea treatment. Vesicles were allowed for recovery in conditioned medium supplemented with EGF, and CI-1033 was added into the medium to a final concentration of 10 μM immediately after the initial EdU pulse. Germinative cell proliferation was analyzed by EdU-BrdU dual labeling after 4 days of recovery. Images show rare EdU^+^ and EdU^+^BrdU^+^ cells following CI-1033 treatment (see the text). (C) Representative images of the accumulations of EdU^+^ germinative cells in some cell aggregates (indicated by the dashed-line boxes) in the vesicles treated with DMSO (control) or 10 μM CI-1033 for 6 days (red: EdU; blue: DAPI). (D) Effects of CI-1033 on the larval growth and development. Vesicles or protoscoleces were cultivated in DMSO-containing conditioned medium (control) supplemented with the ingredients as indicated. Vesicle growth (left) and vesicle formation from protoscoleces (right) were analyzed after 28 days and 18 days of cultivation, respectively. Data are shown as mean ± SD of triplicates, representative of 3 independent experiments. ** P < 0.01 and *** P < 0.001. Bar = 20 μm in (A), (B) and (C).

We wondered if the impaired EGFR activation would impact *E*. *multilocularis* germinative cell behaviors. To this end, we treated vesicles with CI-1033. The results show that CI-1033 significantly reduced the number of EdU^+^ cells in the vesicles (Fig 4A). Another EGFR inhibitor, BIBW2992 (afatinib) [40], also exhibited a similar effect to CI-1033 on the germinative cells (S5A Fig).

In the EdU-BrdU dual labeling experiments, vesicles were allowed to recover from the hydroxyurea treatment with addition of EGF, and CI-1033 was administrated to the vesicles immediately after the initial EdU pulse. At the end of the labeling period, only 1.6‰ of total cells were EdU^+^ (11 EdU^+^ / 6737 DAPI nuclei), and the EdU^+^BrdU^+^ cells were hardly detected (Fig 4B).

There is a strong accumulation of EdU^+^ cells in numerous aggregates in some developing vesicles, which indicates that the active proliferation and extensive self-renewal of germinative cells may drive the development of brood capsule and protoscolex in the vesicles [15]. Our studies show that CI-1033 can also abolish the accumulation of EdU^+^ cells in these cell aggregates (Fig 4C). Further investigations showed that CI-1033 and BIBW2992 can significantly inhibit the larval growth and development upon EGF stimulation (Fig 4D and S5B Fig).

These results suggest that an EGFR-dependent signaling in the parasite is required for the promoted germinative cell proliferation and larval growth, and that the signaling could probably be impaired by the EGFR inhibitors initially designed against human EGF receptors.

**Fig 5:**
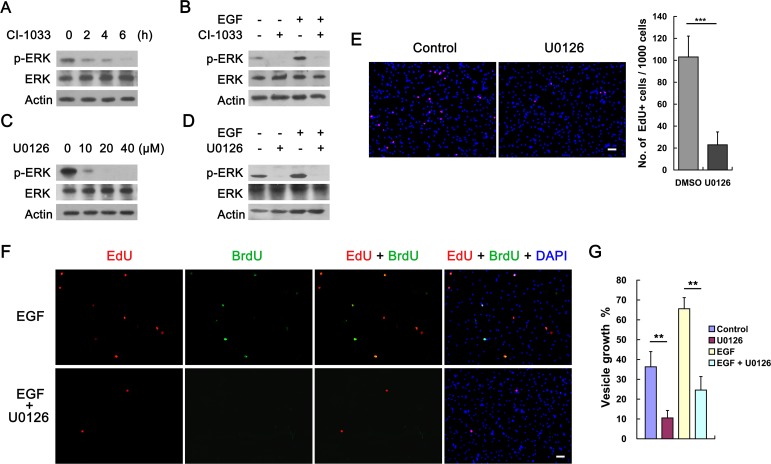
EGFR/ERK signaling contributes to *E. multilocularis* germinative cell proliferation. (A) CI-1033 impairs *E*. *multilocularis* ERK phosphorylation. Vesicles were treated with 10 μM CI-1033 for indicated time. (B) CI-1033 inhibits EGF-stimulated *E*. *multilocularis* ERK phosphorylation. Vesicles were treated with 10 μM CI-1033 (+) or DMSO (-) for 6 h, followed by stimulation with EGF (+) or not (-) for 20 minutes. (C) U0126 impairs *E*. *multilocularis* ERK phosphorylation. Vesicles were treated with indicated concentrations of U0126 for 6 h. (D) U0126 inhibits EGF-stimulated *E*. *multilocularis* ERK phosphorylation. Vesicles were treated with 20 μM U0126 (+) or DMSO (-) for 6 h, followed by stimulation with EGF (+) or not (-) for 20 minutes. (E) U0126 reduces the number of EdU^+^ germinative cells. Vesicles were treated with 20 μM U0126 for 6 days. Representative images are shown on the left and the quantification is shown on the right. Values represent the mean ± SD of 4 separate labeling experiments. *** *P* < 0.001. Bar = 20 μm. (F) Effects of U0126 on germinative cell proliferation during the recovery from hydroxyurea treatment. Vesicles were allowed for recovery in conditioned medium supplemented with EGF and U0126 was added into the medium to a final concentration of 20 μM immediately after the initial EdU pulse. Germinative cell proliferation was analyzed after 4 days of recovery. Images show rare EdU^+^ and EdU^+^BrdU^+^ cells following U0126 treatment (see the text). Bar = 20 μm. (G) Effects of U0126 on the larval growth. Vesicles were incubated with 10 μM U0126 and parasite growth was analyzed after 28 days of cultivation. Data are shown as mean ± SD of triplicates. ** *P* < 0.01.

In *E*. *multilocularis*, structural and functional homologues to mammalian MAP kinase cascade molecules, such as RAF, MEK and ERK, have been identified and characterized [27]. Previous study has shown that host EGF could induce *E*. *multilocularis* ERK activation [32]. Our data show that the basal level of ERK phosphorylation in the vesicles was down-regulated following CI-1033 treatment in a time-dependent manner (Fig 5A). We also found that CI-1033 can inhibit the phosphorylation of ERK induced by EGF (Fig 5B). These results suggest that the EGFR inhibitors could impair the activations of parasite’s EGFR and ERK.

We then treated vesicles with U0126, a MEK inhibitor which effectively suppressed both of the basal and the addition of EGF-induced ERK phosphorylations in the parasite (Fig 5C and 5D). Along with the inhibition of MEK/ERK activity, a remarkable decrease in the number of EdU^+^ germinative cells was observed in the vesicles (Fig 5E). EdU-BrdU dual labeling experiments also indicate that U0126 has a comparable inhibition effect to CI-1033 on the EGF-promoted germinative cell proliferation (2.9‰ of the total cells were EdU^+^ and the EdU^+^BrdU^+^ cells were hardly detected) (Fig 5F). Our further investigation shows that U0126 can significantly inhibit the EGF-stimulated vesicle growth (Fig 5G).

Taken together, these results suggest that the EGF-mediated EGFR/MEK/ERK signaling contributes to germinative cell proliferation during *E*. *multilocularis* larval growth and development.

## Discussion

Throughout the complex life cycle of *E*. *multilocularis*, the parasite always keeps a population of stem cell-like cells, the germinative cells, which are considered as one of the fundamental underpinnings of its growth and development in the host [15]. Stem cell maintenance and functions are strictly controlled by signals from the local tissue microenvironments known as “niches”, which have been widely characterized and elucidated for mammals and invertebrate model animals [18]. Proliferation and differentiation of neoblasts, the stem cells in the free-living flatworm planarian, are also regulated by signals from the surrounding cells [11]. However, the situation is somewhat different for the host liver tissue-dwelling metacestode larvae of *E*. *multilocularis*. Due to the intimate parasite-host contact, the parasite is believed to be able to sense signals derived not only from its own tissue but also from the host-derived hormones and cytokines, as these signaling receptors and downstream signaling cascades are evolutionarily conserved between the mammalian hosts and *E*. *multilocularis* [27]. Thus it is tempting to suggest that *E*. *multilocularis* germinative cells should be regulated by the host-derived factors [30].

In the present study, we show that germinative cell proliferation of the *in vitro*-cultivated *E*. *multilocularis* larvae is promoted upon the addition of human EGF, which in turn drives vesicle growth and vesicle formation from protoscolex (Figs 1 and 2). These results suggest that the germinative cells are regulated by the signaling pathways within the parasite that could sense the host-derived EGF signal. A physiologically relevant concentration of EGF (1 ng/mL) showed a modest effect on the growth of *in vitro*-cultivated vesicles, while 10 ng/mL or higher concentrations of EGF greatly stimulated the growth (S1 Fig). We then used 100 ng/mL of EGF, which exhibited the most significant effect on parasite’s growth, for further *in vitro* studies. This concentration of EGF is considerable and widely used in human cancer cell research, and is relevant to those used in the *in vitro* studies of *E*. *multilocularis* and *S*. *mansoni* [32, 37]. However, it could exceed the physiological concentrations in liver. Considering that the larval development of *E*. *multilocularis* causes host liver tissue destruction and regeneration while EGF is continually made available to the liver and strongly produced during regeneration processes [41–42], it will be interesting to investigate the effects of host EGF observed in this study on the parasite in future using *in vivo* infection models.

The promoted proliferation of germinative cells upon EGF stimulation was supported by our EdU-BrdU dual labeling experiments (Fig 2B and 2C and S3 Fig), which also suggest that the cell-cycle time for most of the actively proliferating germinative cells is less than three days (S2 Fig and Fig 2B), similar to that for *S*. *mansoni* adult somatic stem cells [14]. It has been suggested that there are subpopulations of the germinative cells capable of maintaining their pluripotency and self-renewing like stem cells [15]. Although our data suggest that the increased number of EdU^+^BrdU^+^ cells (as well as the increased ratio of EdU^+^BrdU^+^ cells to EdU^+^ cells) may also result from the promoted self-renewal of these stem-like cell populations, due to the limitations of the dual labeling experiments we still could not distinguish the self-renewing cells from the transit amplifying cells. Specific molecular markers of the stem-like cell populations that work independently of proliferation would be needed for further explorations to clarify the contribution of EGF to germinative cell self-renewal in *E*. *multilocularis*.

Addition of host EGF promotes germinative cell proliferation, which then promotes *in vitro*-cultivated protoscoleces to form metacestode vesicles (Fig 1C). The formation of vesicle from protoscolex occurs *in vivo* following the rupture of parasite cysts and distribution of protoscoleces, and is thought to contribute to prolonged parasite survival in the intermediate host [19, 28], which would result in a poor prognosis after surgery-induced rupture of parasite cysts in human echinococcosis, at least in cystic echinococcosis (CE). Interestingly, we found that addition of host EGF may not only promote this formation process but also initiate it by triggering activation of the germinative cells from a quiescent state in the developed protoscoleces (S6 Fig). Koziol *et al*. [15] indicated that there is a large population of germinative cells capable of proliferation in the developed protoscolex, but they remain in a quiescent state or with slow cell-cycle kinetics for as long as the protoscolex remains resting within the metacestode. These quiescent germinative cells were activated when the protoscoleces were activated by artificially mimicking the ingestion by the definitive host or when the protoscoleces were *in vitro* cultured in serum-containing DMEM. Thus it is tempting to suggest that host factors activate the quiescent germinative cells to re-enter the cell-cycle for proliferation and self-renewal, which may further stimulate the protoscoleces to mature into adults within the definitive host’s intestine or to form metacestode vesicles in the intermediate host’s liver and other tissues. It is still unclear how protoscoleces alternate between developmental fates: the adult or metacestode vesicle. In any case, this unique development potential for protoscoleces is based on the germinative cells, which may response to different host-derived signals from different host tissue microenvironments. Our data suggest that host factors may play a vital role in host-parasite interaction via mediating the relative signaling pathways in the parasite to regulate germinative cell functions.

It has been suggested that host EGF could activate the highly conserved Ras/Raf/MEK/ERK signaling cascade in *E*. *multilocularis*, which is probably mediated by the parasite’s EGF-receptor-like kinase [31–33]. Besides EmER, the first EGFR homologue identified in *E*. *multilocularis* [31], two additional members of the EGFR family could be identified, which display significant homologies with human EGFR and *S*. *mansoni* EGFR in the functional domains (S4A–S4D Fig). Like EmER, these two EGFR homologues are continually expressed in *E*. *multilocularis* metacestode vesicles and protoscoleces (S4E Fig). It will be interesting to clarify their roles as the EGF receptor kinase in the parasite’s development within the host in the future work. In this study, using the *Xenopus* oocyte expression system we show that one of the EGFR homologues EmER can be activated by host EGF (Fig 3). We also show here that inhibition of the MEK/ERK signaling activation by either the EGFR inhibitors CI-1033 and BIBW2992 or the MEK inhibitor U0126 significantly impaired *E*. *multilocularis* germinative cell proliferation, larval growth and development (Figs 4 and 5 and S5 Fig). In mammals, the MEK/ERK pathway plays a critical role in regulating stem cells. For example, it is required for maintenance of stemness and self-renewal of mouse neural stem/precursor cells [43–44]. The role of MEK/ERK pathway in mammalian embryonic stem cells (ESCs) is much more complex. The MEK/ERK signaling plays a functional role in promoting differentiation of mouse ESCs, while it promotes self-renewal of human ESCs (reviewed in [20] and [45]). In invertebrates, much of what is known about the role of the MEK/ERK signaling in regulation of stem cells derives from the studies of *Drosophila*. It has been extensively documented that the EGFR-dependent activation of the MEK/ERK signaling pathway is essential for promoting the maintenance and self-renewal of various types of adult somatic stem cells in *Drosophila* [23–24, 46]. Our data also show that EGF-promoted germinative cell proliferation and larval growth rely on the activation of the parasite’s EGFR/ERK signaling. However, it still remains unclear that the contribution of EGFR/ERK signaling to the promoted proliferation is attributed to the direct response in the germinative cells or to the indirect response to a second signal produced by their surrounding differentiated cells upon EGF stimulation, or to both. Further analysis of the EGFR activation in proliferating germinative cells would be needed to clarify this issue.

While downstream of EGFR lies the PI3K/AKT, MEK/ERK and STAT3 pathways, our findings define here that the MEK/ERK pathway contributes to the role of EGFR signaling in regulating *E*. *multilocularis* germinative cell proliferation. Considering that the PI3K/AKT pathway in *E*. *multilocularis* was recently suggested to be involved in the host insulin-stimulated germinative cell proliferation [28], we also treated metacestode vesicles with the PI3K inhibitor LY294002, which was shown to effectively inhibit *E*. *multilocularis* PI3K activity [28]. We found that LY294002 did not exhibit as obvious an inhibitory effect as U0126 on the proliferation of germinative cells but slightly decreased the number of EdU^+^ cells in metacestode vesicles. Future work would be required for evaluating the contribution of EGFR/AKT/PI3K signaling to the regulation of *E*. *multilocularis* germinative cells.

Increasing evidence has shown that the inhibitors originally designed against the human kinases can effectively inhibit the activity of related kinases in *E*. *multilocularis* [28, 47–49]. Based on the evolutionary conservation among the kinases of vertebrates and invertebrates (including invertebrate parasites), it has been widely considered that small molecules that target human kinases are promising drug candidates for treating human helminthiasis, including echinococcosis [50–51]. Considering our observations that both basal ERK phosphorylation and ERK phosphorylation induced by administered EGF were effectively suppressed by either CI-1033 or U0126 (Fig 5A–5D), it is therefore conceivable that the kinase inhibitors used in this study could impair EGFR/ERK signaling in *E*. *multilocularis*. Although these inhibitors were used within the range concentrations required for these compounds to specifically inhibit their respective targets in humans, it is possible that they may also have cellular targets other than EGFR/ERK signaling in *E*. *multilocularis*.

Our findings suggest that exogenous EGF-activated EGFR/ERK signaling in the parasite was inhibited by CI-1033. Given that the long-term *in vitro* maintenance of *E*. *multilocularis* larvae and primary cells requires continuous presence of host cell-derived growth factors [29], it is reasonable to assume that EGFR inhibitors could impair the activation of parasite’s EGFR upon host EGF stimulation, which might be the main reason for the diminished germinative cell proliferation and the impaired larval growth and development (Fig 4 and S5 Fig). However, our data could not exclude the possibility that EGFR inhibitors may also impair the parasite’s EGFR activation mediated by its own EGF molecules. It has been recently shown that neoblast clonal expansion in the free-living flatworm planarian is regulated by its own EGF-mediated EGFR signaling [52]. Since the parasite also possesses a putative EGF homologue [53], this endogenous EGFR ligand-mediated signaling might also be involved in regulating *E*. *multilocularis* germinative cells. Improved *in vitro* cultivation systems and methods that could avoid/reduce the impacts of host factors will be helpful to investigate the roles of this endogenous signaling in germinative cell regulation.

Although stem cell-like germinative cells has been widely described in tapeworms and their roles in the parasite’s development within the host are thought to be of fundamental importance, there are still long standing gaps in our knowledge of mechanisms controlling the behavior of these cells. This study defines an essential role for the EGF-mediated EGFR/ERK signaling in promoting germinative cell proliferation in *E*. *multilocularis*. It makes an effort to unravel the mechanisms of regulation of tapeworm germinative cells in response to host-derived growth factors, and helps in understanding the delicate developmental strategies of these parasites within the host. Targeting the signaling pathways involved in regulating germinative cells may provide a novel therapeutic strategy against echinococcosis and other human cestodiasis.

## Supporting information

S1 Fig Effects of EGF on *E. multilocularis* larval growth. Metacestode vesicles were cultivated in conditioned medium (Control) supplemented with 1–100 ng/mL recombinant human EGF for 49 days. Vesicle growth is shown as the increase of vesicle diameter as compared to day 0 for each group. Data are shown as mean ± SD of triplicates, representative of two independent experiments.
(TIF)

S2 Fig EdU-BrdU dual labeling experiments. (A) EdU labeling and phospho-Histone H3 (Ser10, p-H3) immunofluorescence. Metacestode vesicles were administrated to a 4-h EdU pulse, and after 44 hours of pulse about 88% (365/413) of p-H3^+^ mitotic cells are EdU^+^. The chase period of 44 h was then used for EdU-BrdU dual labeling experiments. Note the low percentage of p-H3^+^ cells, which is consistent with a previous report [15]. (B) Analysis of proliferating germinative cells by EdU labeling in hydroxyurea (HU) treatment experiments. Metacestode vesicles were treated with 40 mM of hydroxyurea for three days and then allowed for recovery in conditioned medium. Representative images are shown as: no treatment control, hydroxyurea treatment, and 4 days of recovery after removal of hydroxyurea (red: EdU; blue: DAPI). Bar = 40 μm. (C) Timeline for hydroxyurea treatment and EdU-BrdU dual labeling. Metacestode vesicles were pretreated with 40 mM of hydroxyurea for three days. EGF was immediately added into the conditioned medium after removal of hydroxyurea. Sequential pulses of EdU and BrdU began at 96 h after removal of hydroxyurea. Dual labeling under normal culture conditions (related to Fig 2B) was carried out without hydroxyurea treatment, which is: EdU label for 4 hours, no label for 44 hours, and BrdU label for 24 hours.
(TIF)

S3 Fig EdU^+^BrdU^+^ cells are highly presented in the clonally proliferating germinative cells. Metacestode vesicles were treated with 40 mM hydroxyurea (HU) for seven days and then transferred to HU-free medium. Samples were administrated to EdU-BrdU dual labeling at the day 3 after HU removal. Dashed line boxes in (A) indicate patches of EdU^+^ cells clonally growing. Bar = 100 μm. The magnified views are shown as in (B). Bar = 20 μm.
(TIF)

S4 Fig Analysis of amino acid sequence and mRNA expression of the EGF receptor members of *E. multilocularis*. (A)-(C) Amino acid sequence analysis of the receptor-L-domain 1 (A), receptor-L-domain 2 (B) and Pkinase _Tyr domain (C) of human (Hs), *S*. *mansoni* (Sm) and *E*. *multilocularis* (Em) EGF receptors. Domains are predicted using the online software (http://scansite3.mit.edu/). Positions at which all of the residues are conserved are shaded in black. (D) Similarities of *E*. *multilocularis* EGF receptor members to human EGFR. Similarity values to the L-C-L domain (two receptor L domains separated by a cysteine-rich furin-like region) and the kinase domain are indicated below as % identical residues (not bracketed) and % similar residues (bracketed). Further indicated are the similarities of overall protein sequences. (E) RT-PCR analysis of mRNA expression of *E*. *multilocularis* EGF receptor members in protoscoleces (lane 1–3) and metacestode vesicles (lane 4–6). Lane 1 and 4: EmER. Lane 2 and 5: Em_000617300. Lane 3 and 6: Em_000969600. M indicates the DNA marker.
(TIF)

S5 Fig BIBW2992 impairs *E. multilocularis* germinative cell proliferation, larval growth and development. (A) Representative images of EdU^+^ germinative cells in the metacestode vesicles following treatment of 5 μM BIBW2992 or DMSO control for 3 days (red: EdU; blue: DAPI). Bar = 20 μm. (B) Effects of BIBW2992 on the larval growth and development. Vesicles or protoscoleces were cultivated in the DMSO-containing conditioned medium (control) supplemented with the ingredients as indicated. Vesicle growth (left) and vesicle formation from protoscoleces (right) were analyzed after 28 days and 18 days of cultivation, respectively. Data are shown as mean ± SD of triplicates, representative of 2–3 independent experiments. *** *P* < 0.001.
(TIF)

S6 Fig EGF stimulates the quiescent germinative cells in the developed protoscoleces. Protoscoleces freshly isolated from the metacestode material were *in vitro* maintained in PBS supplemented with EGF or not for 12h followed by a 4-hour pulse of EdU. Few EdU^+^ cells presented in the developed protoscoleces (arrows), however, the number of EdU^+^ cells dramatically increased after EGF stimulation. The arrow head indicates a developing protoscolex which possesses plenty of EdU^+^ cells. Bar = 100 μm.
(TIF)

## References

[1] Ladurner P, Rieger R, Baguna J. Spatial distribution and differentiation potential of stem cells in hatchlings and adults in the marine *Platyhelminth macrostomum* sp.: a bromodeoxyuridine analysis. Dev Biol. 2000; 226: 231–241. 10.1006/dbio.2000.9867 11023683

[2] S´anchez Alvarado A, Newmark PA, Robb SM, Juste R. The *Schmidtea mediterranea* database as amolecular resource for studying platyhelminthes, stem cells and regeneration. Development. 2002; 129: 5659–5665. 12421706 10.1242/dev.00167

[3] Reuter M, Kreshchenko N. Flatworm asexual multiplication implicates stem cells and regeneration. Can J Zool. 2004; 82: 334–356.

[4] De Mulder K, Kuales G, Pfister D, Willems M, Egger B, Salvenmoser W, et al Characterization of the stem cell system of the acoel *Isodiametra pulchra*. BMC Dev Biol. 2009; 9: 69 10.1186/1471-213X-9-69 20017953 PMC2806412

[5] Shibata N, Rouhana L, Agata K. Cellular and molecular dissection of pluripotent adult somatic stem cells in planarians. Dev Growth Differ. 2010; 52: 27–41. 10.1111/j.1440-169X.2009.01155.x 20078652

[6] Peter R, Gschwentner R, Schürmann W, Rieger RM, Ladurner P. The significance of stem cells in free-living flatworms: one common source for all cells in the adult. J Appl Biomed. 2004; 2: 21–35.

[7] Pellettieri J, Sanchez AA. Cell turnover and adult tissue homeostasis: from humans to planarians. Annu Rev Genet. 2007; 41: 83–105. 10.1146/annurev.genet.41.110306.130244 18076325

[8] Eisenhoffer GT, Kang H, Sanchez Alvarado A. Molecular analysis of stem cells and their descendants during cell turnover and regeneration in the planarian *Schmidtea mediterranea*. Cell Stem Cell. 2008; 3: 327–339. 10.1016/j.stem.2008.07.002 18786419 PMC2614339

[9] Pfister D, De Mulder K, Hartenstein V, Kuales G, Borgonie G, Marx F, et al Flatworm stem cells and the germ line: developmental and evolutionary implications of macvasa expression in *Macrostomum lignano*. Dev Biol. 2008; 319: 146–159. 10.1016/j.ydbio.2008.02.045 18405892

[10] Wagner DE, Wang IE, Reddien PW. Clonogenic neoblasts are pluripotent adult stem cells that underlie planarian regeneration. Science. 2011; 332: 811–816. 10.1126/science.1203983 21566185 PMC3338249

[11] Rink JC. Stem cell systems and regeneration in planaria. Dev. Genes Evol. 2013; 223: 67–84. 10.1007/s00427-012-0426-4 23138344 PMC3552358

[12] Koziol U, Domínguez MF, Marín M, Kun A, Castillo E. Stem cell proliferation during in vitro development of the model cestode *Mesocestoides corti* from larva to adult worm. Front Zool. 2010; 7: 22 10.1186/1742-9994-7-22 20626875 PMC2917415

[13] Wang B, Collins JJ 3rd, Newmark PA. Functional genomic characterization of neoblast-like stem cells in larval *Schistosoma mansoni*. Elife. 2013; 2: e00768 10.7554/eLife.00768 23908765 PMC3728622

[14] Collins JJ 3rd, Wang B, Lambrus BG, Tharp ME, Iyer H, Newmark PA. Adult somatic stem cells in the human parasite *Schistosoma mansoni*. Nature. 2013; 494: 476–479. 10.1038/nature11924 23426263 PMC3586782

[15] Koziol U, Rauschendorfer T, Zanon Rodriguez L, Krohne G, Brehm K. The unique stem cell system of the immortal larva of the human parasite *Echinococcus multilocularis*. Evodevo. 2014; 5: 10 10.1186/2041-9139-5-10 24602211 PMC4015340

[16] Brehm K. *Echinococcus multilocularis* as an experimental model in stem cell research and molecular host-parasite interaction. Parasitology. 2010; 137: 537–555. 10.1017/S0031182009991727 19961652

[17] Eckert J, Deplazes P. Biological, epidemiological, and clinical aspects of echinococcosis, a zoonosis of increasing concern. Clin Microbiol Rev. 2004; 17: 107–135. 10.1128/CMR.17.1.107-135.2004 14726458 PMC321468

[18] Morrison SJ, Spradling AC. Stem cells and niches: mechanisms that promote stem cell maintenance throughout life. Cell. 2008; 132: 598–611. 10.1016/j.cell.2008.01.038 18295578 PMC4505728

[19] Hackett JA, Surani MA. Regulatory principles of pluripotency: from the ground state up. Cell Stem Cell. 2014; 15: 416–430. 10.1016/j.stem.2014.09.015 25280218

[20] Huang G, Ye S, Zhou X, Liu D, Ying QL. Molecular basis of embryonic stem cell self-renewal: from signaling pathways to pluripotency network. Cell Mol Life Sci. 2015; 72: 1741–1757. 10.1007/s00018-015-1833-2 25595304 PMC4809369

[21] Ferron SR, Pozo N, Laguna A, Aranda S, Porlan E, Moreno M, et al Regulated segregation of kinase Dyrk1A during asymmetric neural stem cell division is critical for EGFR-mediated biased signaling. Cell Stem Cell. 2010; 7: 367–379. 10.1016/j.stem.2010.06.021 20804972

[22] Xu N, Wang SQ, Tan D, Gao Y, Lin G, Xi R. EGFR, Wingless and JAK/STAT signaling cooperatively maintain *Drosophila* intestinal stem cells. Dev Biol. 2011; 54: 31–43. 10.1016/j.ydbio.2011.03.018 21440535

[23] Jiang H, Grenley MO, Bravo MJ, Blumhagen RZ, Edgar BA. EGFR/Ras/MAPK signaling mediates adult midgut epithelial homeostasis and regeneration in *Drosophila*. Cell Stem Cell. 2011; 8: 84–95. 10.1016/j.stem.2010.11.026 21167805 PMC3021119

[24] Castanieto A, Johnston MJ, Nystul TG. EGFR signaling promotes self-renewal through the establishment of cell polarity in *Drosophila* follicle stem cells. Elife. 2014; 3: e04437. 10.7554/eLife.04437 PMC4298699 25437306

[25] Jin Y, Ha N, Forés M, Xiang J, Gläßer C, Maldera J, et al EGFR/Ras Signaling Controls *Drosophila* Intestinal Stem Cell Proliferation via Capicua-Regulated Genes. PLoS Genet. 2015; 11: e1005634 10.1371/journal.pgen.1005634 26683696 PMC4684324

[26] Brehm K, Spiliotis M. The influence of host hormones and cytokines on *Echinococcus multilocularis* signalling and development. Parasite. 2008; 15: 286–290. 10.1051/parasite/2008153286 18814696

[27] Brehm K. The role of evolutionarily conserved signalling systems in *Echinococcus multilocularis* development and host-parasite interaction. Med Microbiol Immunol. 2010; 199: 247–259. 10.1007/s00430-010-0154-1 20376483

[28] Hemer S, Konrad C, Spiliotis M, Koziol U, Schaack D, Forster S, et al Host insulin stimulates *Echinococcus multilocularis* insulin signalling pathways and larval development. BMC Biology. 2014; 12: 5 10.1186/1741-7007-12-5 24468049 PMC3923246

[29] Brehm K, Spiliotis M. Recent advances in the in vitro cultivation and genetic manipulation of *Echinococcus multilocularis* metacestodes and germinal cells. Exp Parasitol. 2008; 119: 506–515. 10.1016/j.exppara.2008.03.007 18439582

[30] Koziol U, Brehm K. Recent advances in *Echinococcus* genomics and stem cell research. Vet Parasitol. 2015; 213: 92–102. 10.1016/j.vetpar.2015.07.031 26296590

[31] Spiliotis M, Kroner A, Brehm K. Identification, molecular characterization and expression of the gene encoding the epidermal growth factor receptor orthologue from the fox-tapeworm *Echinococcus multilocularis*. Gene. 2003; 323: 57–65. 14659879 10.1016/j.gene.2003.09.007

[32] Spiliotis M, Konrad C, Gelmedin V, Tappe D, Bruckner S, Mosch HU, et al Characterisation of EmMPK1, an ERK-like MAP kinase from *Echinococcus multilocularis* which is activated in response to human epidermal growth factor. Int J Parasitol. 2006; 36: 1097–1112. 10.1016/j.ijpara.2006.05.008 16793045

[33] Gelmedin V, Spiliotis M, Brehm K. Molecular characterisation of MEK1/2- and MKK3/6-like mitogen-activated protein kinase kinases (MAPKK) from the fox tapeworm *Echinococcus multilocularis*. Int J Parasitol. 2010; 40: 555–567. 10.1016/j.ijpara.2009.10.009 19887070

[34] Tang CT, Quian YC, Kang YM, Cui GW, Lu HC, Shu LM, et al Study on the ecological distribution of alveolar *Echinococcus* in Hulunbeier Pasture of Inner Mongolia, China. Parasitology, 2004; 128: 187–194. 15030006 10.1017/s0031182003004438

[35] Spiliotis M, Brehm K. Axenic *in vitro* cultivation of *Echinococcus multilocularis* metacestode vesicles and the generation of primary cell cultures. Methods Mol Biol. 2009; 470: 245–262. 10.1007/978-1-59745-204-5_17 19089387

[36] Cheng Z, Liu F, Zhu S, Wang L, Wang Y. A rapid and convenient method for fluorescence analysis of *in vitro* cultivated metacestode vesicles from *Echinococcus multilocularis*. PLoS ONE. 2015; 10: e0118215 10.1371/journal.pone.0118215 25705880 PMC4337908

[37] Vicogne J, Cailliau K, Tulasne D, Browaeys E, Yan YT, Fafeur V, et al Conservation of epidermal growth factor receptor function in the human parasitic helminth *Schistosoma mansoni*. J Biol Chem. 2004; 279: 37407–37414. 10.1074/jbc.M313738200 15231836

[38] Salic A, Mitchison TJ. A chemical method for fast and sensitive detection of DNA synthesis *in vivo*. Proc Natl Acad Sci. USA 2008; 105: 2415–2420. 10.1073/pnas.0712168105 18272492 PMC2268151

[39] Allen LF, Eiseman IA, Fry DW, Lenehan PF. CI-1033, an irreversible pan-erbB receptor inhibitor and its potential application for the treatment of breast cancer. Semin Oncol. 2003; 30: 65–78. 14613028 10.1053/j.seminoncol.2003.08.009

[40] Li D, Ambrogio L, Shimamura T, Kubo S, Takahashi M, Chirieac LR, et al BIBW2992, an irreversible EGFR/HER2 inhibitor highly effective in preclinical lung cancer models. Oncogene. 2008; 27: 4702–4711. 10.1038/onc.2008.109 18408761 PMC2748240

[41] Michalopoulos GK, DeFrances MC. Liver regeneration. Science. 1997; 276: 60–66. 9082986 10.1126/science.276.5309.60

[42] Michalopoulos GK. Liver regeneration. J Cell Physiol. 2007; 213: 286–300. 10.1002/jcp.21172 17559071 PMC2701258

[43] Campos LS, Leone DP, Relvas JB, Brakebusch C, Fässler R, Suter U, et al Beta1 integrins activate a MAPK signalling pathway in neural stem cells that contributes to their maintenance. Development. 2004; 131: 3433–3444. 10.1242/dev.01199 15226259

[44] Hu Q, Zhang L, Wen J, Wang S, Li M, Feng R, et al The EGF receptor-sox2-EGF receptor feedback loop positively regulates the self-renewal of neural precursor cells. Stem Cells. 2010; 28: 279–286. 10.1002/stem.246 19882665

[45] Sun Y, Chen CS, Fu J. Forcing stem cells to behave: a biophysical perspective of the cellular microenvironment. Annu Rev Biophys. 2012; 41:519–542. 10.1146/annurev-biophys-042910-155306 22404680 PMC4123632

[46] Li Z, Liu S, Cai Y. EGFR/MAPK signaling regulates the proliferation of *Drosophila* renal and nephric stem cells. J Genet Genomics. 2015; 42: 9–20. 10.1016/j.jgg.2014.11.007 25619598

[47] Gelmedin V, Caballero-Gamiz R, Brehm K. Characterization and inhibition of a p38-like mitogen-activated protein kinase (MAPK) from *Echinococcus multilocularis*: antiparasitic activities of p38 MAPK inhibitors. Biochem Pharmacol. 2008; 76: 1068–1081. 10.1016/j.bcp.2008.08.020 18789902

[48] Schubert A, Koziol U, Calilliau K, Vanderstraete M, Dissous C, Brehm K. Targeting *Echinococcus multilocularis* stem cells by inhibition of the polo-like kinase EmPlk1. PLoS Negl Trop Dis. 2014; 8: e2870 10.1371/journal.pntd.0002870 24901228 PMC4046951

[49] Hemer S, Brehm K. In vitro efficacy of the anticancer drug imatinib on *Echinococcus multilocularis* larvae. Int J Antimicrob Agents. 2012; 40: 458–462. 10.1016/j.ijantimicag.2012.07.007 22947125

[50] Taylor CM, Martin J, Rao RU, Powell K, Abubucker S, Mitreva M. Using existing drugs as leads for broad spectrum anthelmintics targeting protein kinases. PLoS Pathog. 2013; 9: e1003149 10.1371/journal.ppat.1003149 23459584 PMC3573124

[51] Brehm K, Koziol U. On the importance of targeting parasite stem cells in anti-echinococcosis drug development. Parasite. 2014; 21: 72 10.1051/parasite/2014070 25526547 PMC4271656

[52] Lei K, Thi-Kim Vu H, Mohan RD, McKinney SA, Seidel CW, Alexander R, et al Egf signaling directs neoblast repopulation by regulating asymmetric cell division in planarians. Dev Cell. 2016; 38: 413–429. 10.1016/j.devcel.2016.07.012 27523733 PMC5511498

[53] Brehm K, Wolf M, Beland H, Kroner A, Frosch M. Analysis of differential gene expression in *Echinococcus multilocularis* larval stages by means of spliced leader differential display. Int J Parasitol. 2003; 33: 1145–1159. 13678631 10.1016/s0020-7519(03)00169-3

