# Supplementary figures and images for: EGF-mediated EGFR/ERK signaling pathway promotes germinative cell proliferation in Echinococcus multilocularis that contributes to larval growth and development
Source: PLoS Negl Trop Dis. 2017 Feb 27;11(2):e0005418. doi: 10.1371/journal.pntd.0005418 (PMC5344531; doi:10.1371/journal.pntd.0005418)

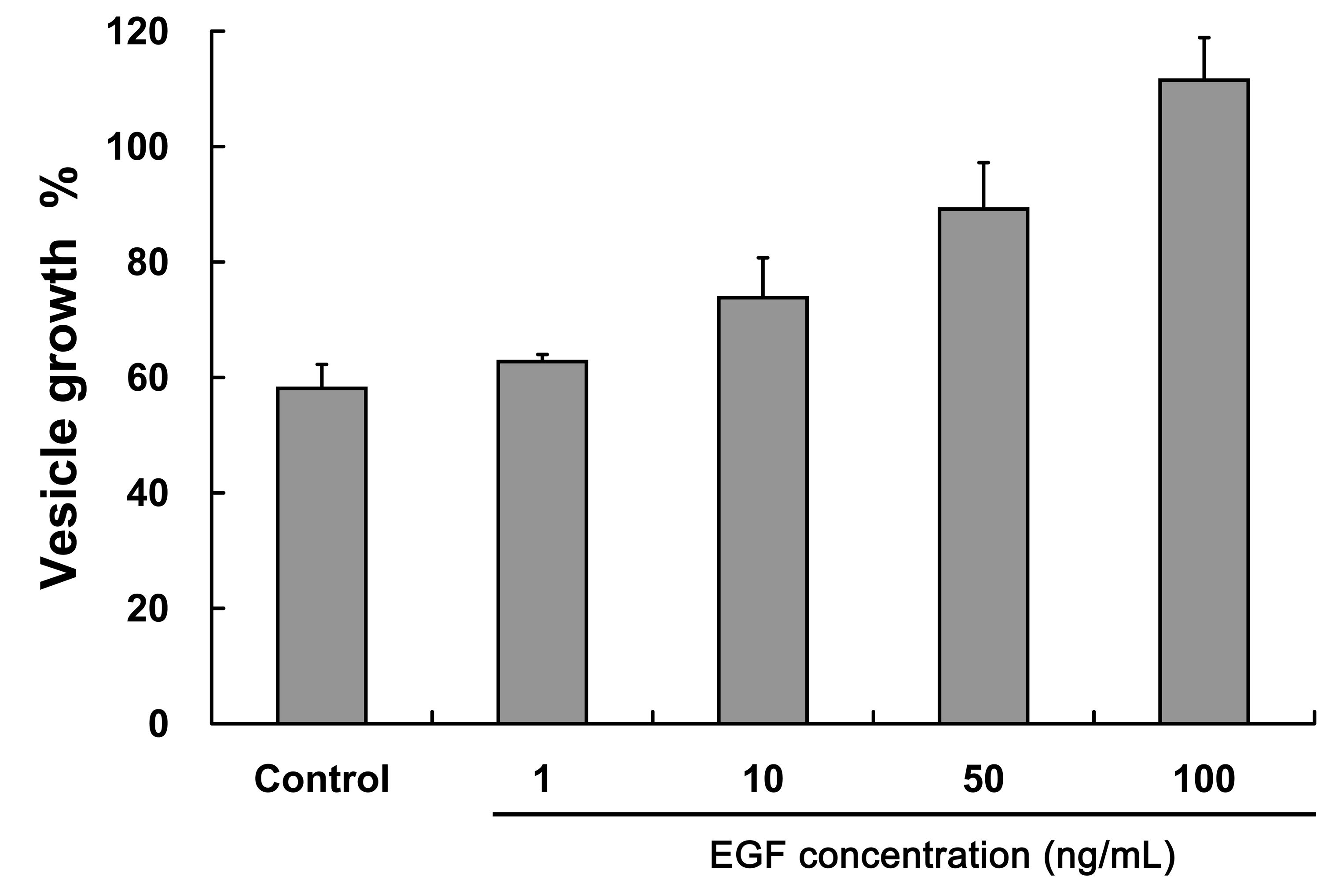

Supplement: S1 Fig — Metacestode vesicles were cultivated in conditioned medium (Control) supplemented with 1–100 ng/mL recombinant human EGF for 49 days. Vesicle growth is shown as the increase of vesicle diameter as compared to day 0 for each group. Data are shown as mean ± SD of triplicates, representative of two independent experiments. (TIF) [file pntd.0005418.s001.tif]

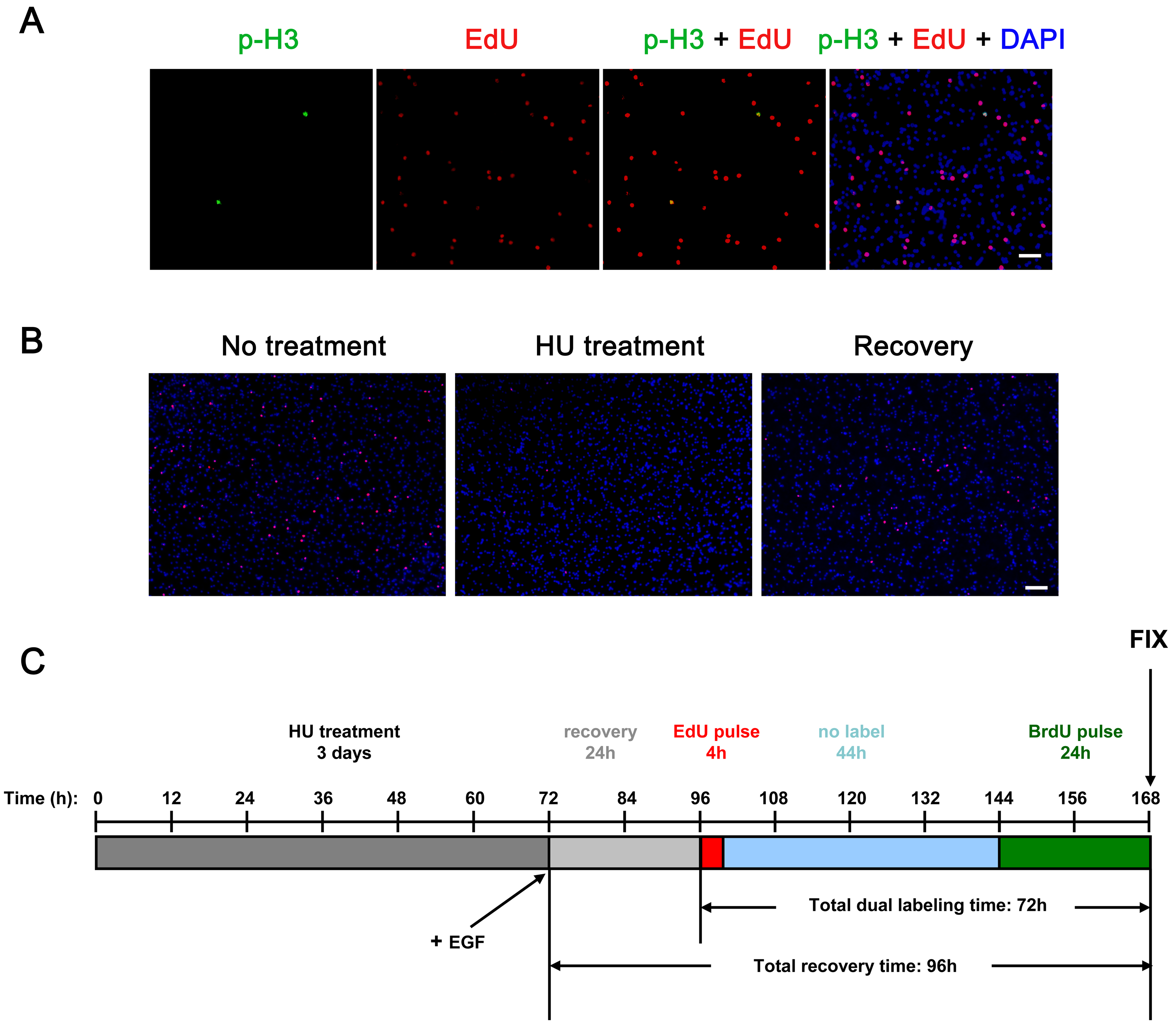

Supplement: S2 Fig — (A) EdU labeling and phospho-Histone H3 (Ser10, p-H3) immunofluorescence. Metacestode vesicles were administrated to a 4-h EdU pulse, and after 44 hours of pulse about 88% (365/413) of p-H3+ mitotic cells are EdU+. The chase period of 44 h was then used for EdU-BrdU dual labeling experiments. Note the low percentage of p-H3+ cells, which is consistent with a previous report [15]. (B) Analysis of proliferating germinative cells by EdU labeling in hydroxyurea (HU) treatment experiments. Metacestode vesicles were treated with 40 mM of hydroxyurea for three days and then allowed for recovery in conditioned medium. Representative images are shown as: no treatment control, hydroxyurea treatment, and 4 days of recovery after removal of hydroxyurea (red: EdU; blue: DAPI). Bar = 40 μm. (C) Timeline for hydroxyurea treatment and EdU-BrdU dual labeling. Metacestode vesicles were pretreated with 40 mM of hydroxyurea for three days. EGF was immediately added into the conditioned medium after removal of hydroxyurea. Sequential pulses of EdU and BrdU began at 96 h after removal of hydroxyurea. Dual labeling under normal culture conditions (related to Fig 2B) was carried out without hydroxyurea treatment, which is: EdU label for 4 hours, no label for 44 hours, and BrdU label for 24 hours. (TIF) [file pntd.0005418.s002.tif]

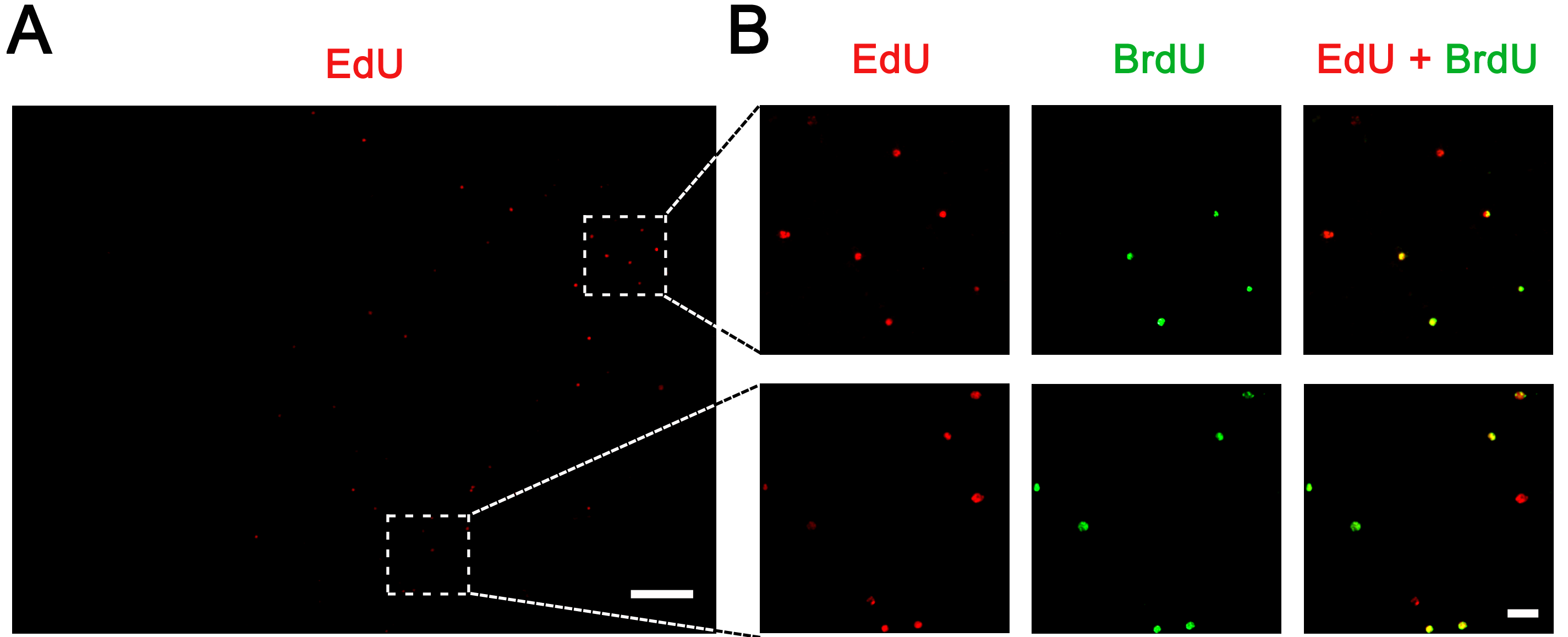

Supplement: S3 Fig — Metacestode vesicles were treated with 40 mM hydroxyurea (HU) for seven days and then transferred to HU-free medium. Samples were administrated to EdU-BrdU dual labeling at the day 3 after HU removal. Dashed line boxes in (A) indicate patches of EdU+ cells clonally growing. Bar = 100 μm. The magnified views are shown as in (B). Bar = 20 μm. (TIF) [file pntd.0005418.s003.tif]

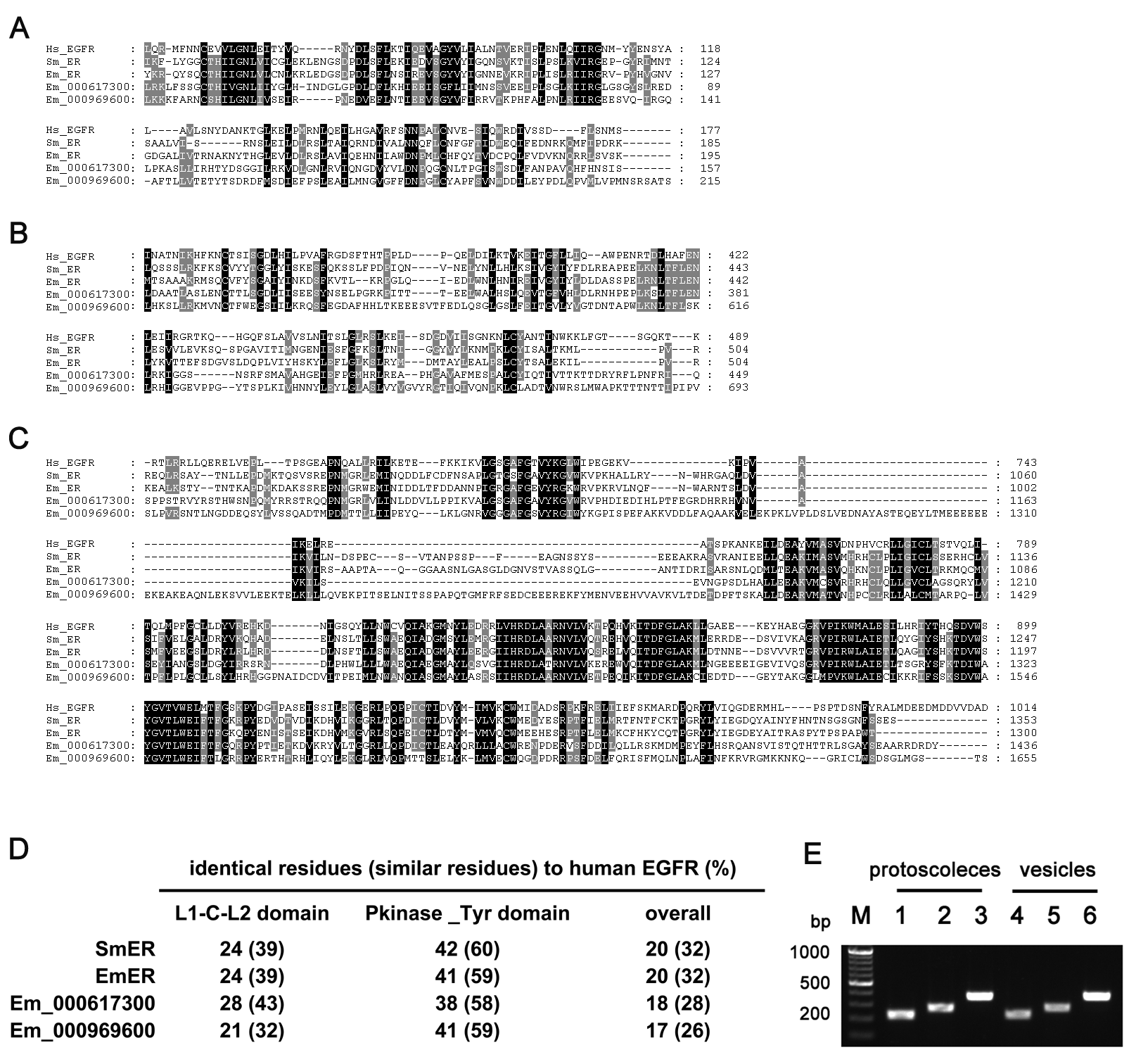

Supplement: S4 Fig — (A)-(C) Amino acid sequence analysis of the receptor-L-domain 1 (A), receptor-L-domain 2 (B) and Pkinase _Tyr domain (C) of human (Hs), S. mansoni (Sm) and E. multilocularis (Em) EGF receptors. Domains are predicted using the online software (http://scansite3.mit.edu/). Positions at which all of the residues are conserved are shaded in black. (D) Similarities of E. multilocularis EGF receptor members to human EGFR. Similarity values to the L-C-L domain (two receptor L domains separated by a cysteine-rich furin-like region) and the kinase domain are indicated below as % identical residues (not bracketed) and % similar residues (bracketed). Further indicated are the similarities of overall protein sequences. (E) RT-PCR analysis of mRNA expression of E. multilocularis EGF receptor members in protoscoleces (lane 1–3) and metacestode vesicles (lane 4–6). Lane 1 and 4: EmER. Lane 2 and 5: Em_000617300. Lane 3 and 6: Em_000969600. M indicates the DNA marker. (TIF) [file pntd.0005418.s004.tif]

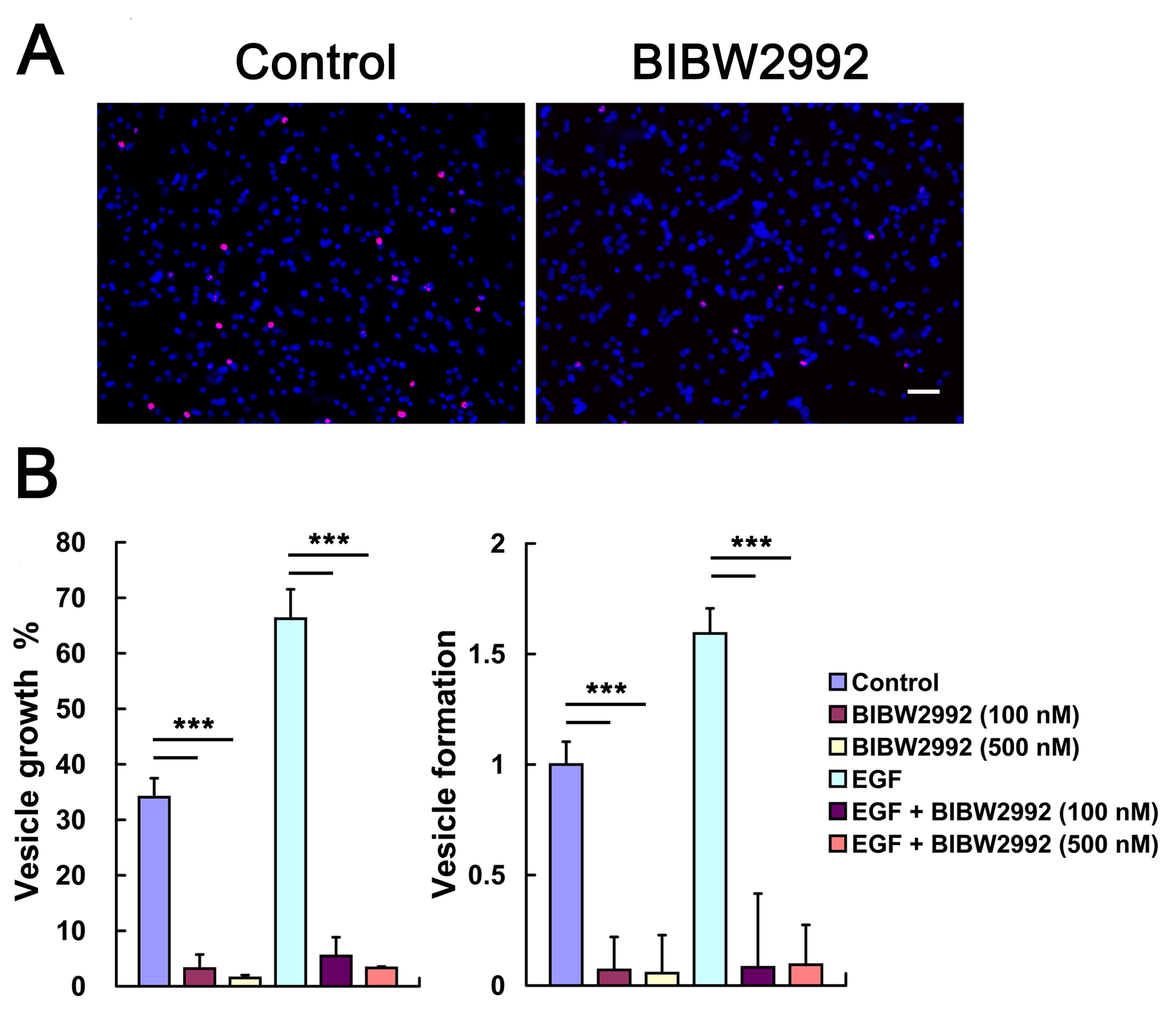

Supplement: S5 Fig — (A) Representative images of EdU+ germinative cells in the metacestode vesicles following treatment of 5 μM BIBW2992 or DMSO control for 3 days (red: EdU; blue: DAPI). Bar = 20 μm. (B) Effects of BIBW2992 on the larval growth and development. Vesicles or protoscoleces were cultivated in the DMSO-containing conditioned medium (control) supplemented with the ingredients as indicated. Vesicle growth (left) and vesicle formation from protoscoleces (right) were analyzed after 28 days and 18 days of cultivation, respectively. Data are shown as mean ± SD of triplicates, representative of 2–3 independent experiments. *** P < 0.001. (TIF) [file pntd.0005418.s005.tif]

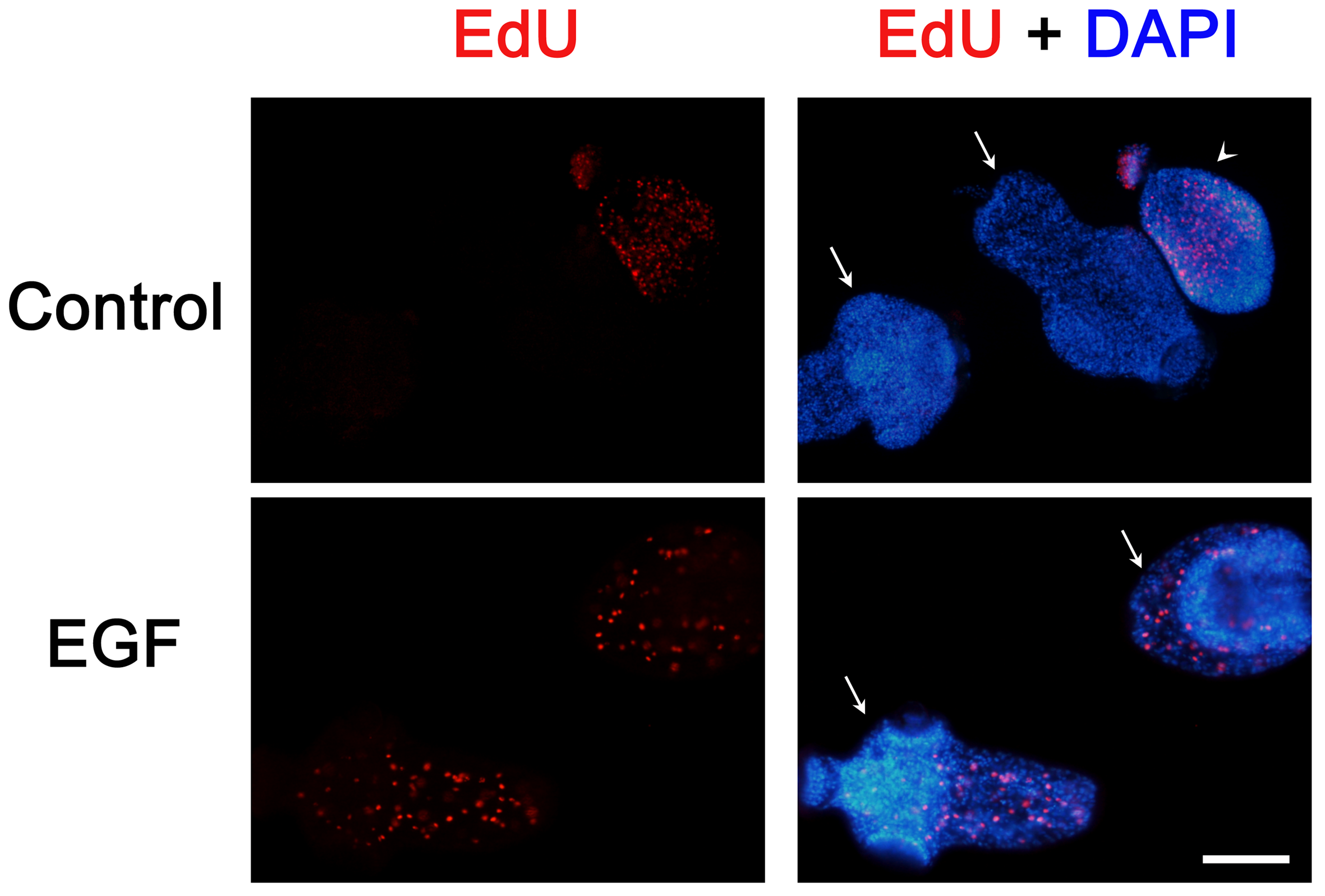

Supplement: S6 Fig — Protoscoleces freshly isolated from the metacestode material were in vitro maintained in PBS supplemented with EGF or not for 12h followed by a 4-hour pulse of EdU. Few EdU+ cells presented in the developed protoscoleces (arrows), however, the number of EdU+ cells dramatically increased after EGF stimulation. The arrow head indicates a developing protoscolex which possesses plenty of EdU+ cells. Bar = 100 μm. (TIF) [file pntd.0005418.s006.tif]
